# Efficacy of the projection onto convex sets (POCS) algorithm at Gd-EOB-DTPA-enhanced hepatobiliary-phase hepatic MRI

**DOI:** 10.1186/s40064-016-2968-9

**Published:** 2016-08-09

**Authors:** Shinichi Nakamura, Takeshi Nakaura, Masafumi Kidoh, Kazuo Awai, Tomohiro Namimoto, Ichiro Yoshinaka, Kazunori Harada, Yasuyuki Yamashita

**Affiliations:** 1 Department of Diagnostic Radiology, Amakusa Regional Medical Center, Amakusa, Japan; 2 Department of Radiology, Kumamoto Rousai Hospital, 1670, Takehara-Machi, Yatushiro, Kumamoto 866-8533 Japan; 3 Department of Diagnostic Radiology, Faculty of Life Sciences, Kumamoto University, Kumamoto, Japan; 4 Diagnostic Radiology, School of Biomedical Sciences, Hiroshima University, Hiroshima, Japan; 5 Department of Surgery, Amakusa Regional Medical Center, Amakusa, Japan

**Keywords:** Projection onto convex sets, Iterative, Hepatobiliary phase, Hepatic MRI, Gd-EOB-DTPA

## Abstract

**Purpose:**

To investigate the efficacy of the projection onto convex sets (POCS) algorithm at Gd-EOB-DTPA-enhanced hepatobiliary-phase MRI.

**Methods:**

In phantom study, we scanned a phantom and obtained images by conventional means (P1 images), by partial-Fourier image reconstruction (PF, P2 images) and by PF with the POCS algorithm (P3 images). Then we acquired and compared subtraction images (P2–P1 images and P3–P1 images). In clinical study, 55 consecutive patients underwent Gd-EOB-DTPA (EOB)-enhanced 3D hepatobiliary-phase MRI on a 1.5T scanner. Images were obtained using conventional method (C1 images), PF (C2 images), and PF with POCS (C3 images). The acquisition time was 17-, 14-, and 14 s for protocols C1, C2 and C3, respectively. Two radiologists assigned grades for hepatic vessel sharpness and we compared the visual grading among the 3 protocols. And one radiologist compared signal-to-noise-ratio (SNR) of the hepatic parenchyma.

**Results:**

In phantom study, there was no difference in signal intensity on a peripheral phantom column on P3–P1 images. In clinical study, there was no significant difference between C1 and C3 images (2.62 ± 0.49 vs. 2.58 ± 0.49, p = 0.70) in the score assigned for vessel sharpness nor in SNR (13.3 ± 2.67 vs. 13.1 ± 2.51, p = 0.18).

**Conclusion:**

The POCS algorithm makes it possible to reduce the scan time of hepatobiliary phase (from 17 to 14 s) without reducing SNR and without increasing artifacts.

## Background

Conventional magnetic resonance imaging (MRI) techniques reconstruct three-dimensional (3D) images from completely sampled k-space data (i.e. a fully sampled, band-limited, spatial frequency space) directly by inverse Fourier transform (Kumar et al. 1975). Fast MRI techniques use strategies to reduce the total scan time (Kruger et al. 2002; Sabati et al. 2003). The partial Fourier (PF) method (Feinberg et al. 1986; Haacke et al. 1990) is another approach to shorten the total scan time. However, the amount of acquired data is reduced due to asymmetric truncation of the peripheral portions of the k-space in either the phase-encoding direction (to reduce N_phase_) (Feinberg et al. 1986) or the frequency-encoding direction (to reduce repetition time; TR) (Haacke et al. 1990), or both. The disadvantage of this technique is image blurring (Chen et al. 2008). The peripheral portion of k-space provides information on only the details and edges of objects in the image domain (Moratal et al. 2008). Blurring of the edge occurs when the phase constraint is insufficient to recover the missing k-space information (Xu and Haacke 2001) and decreases image quality.

Projection onto convex sets (POCS) algorithm is an iterative constrained approach that has been used to reconstruct images from truncated k-space by attempting to extrapolate values for truncated data (Xu and Haacke 2001; Singh et al. 2004). Peng et al. (2006) suggested that this algorithm reduced artifacts compared to partial Fourier imaging and others (Raj et al. 2006) found it useful at MR angiography (MRA). However, the value of the POCS algorithm when used with the 3D volumetric interpolated gradient-echo sequence employed at breath-hold hepatic MRI remains unknown.

Hepatobiliary-phase MRI have scanned at latter period of entire scans. In this situation, breath holding, especially in older patients, might be sometimes difficult. If the image quality of PF with POCS is similar to conventional method, we can replace conventional method of hepatobiliary-phase to PF with POCS and shorten scan time and lighten patient’s burden.

The purpose of this study was to investigate the efficacy of the POCS algorithm about reducing scan time at Gd-EOB-DTPA-enhanced hepatobiliary-phase MRI.

## Methods

### POCS

POCS, developed as an iterative method to reduce blurring (Raj et al. 2006; McGibney et al. 1993), is one of the techniques to improve image quality. It is based on the principle that the correct image is the intersection of all images whose Fourier transform agrees with the measured partial data and of all images whose phase is the same as the phase estimate. To find the intersection of these two image sets, the dataset is first inverse Fourier-transformed into the image domain where the complex values of the image are projected onto a line that is at an angle equal to the phase estimate, i.e.

$$\uprho_{{{\text{y}}.{\text{corrected}}}} \left( {\text{x}} \right) = |\uprho_{\text{y}} \left( {\text{x}} \right)|{ \cos }[\hat{\upphi }_{\text{y}} ({\text{x}}) - \upphi_{\text{y}} \left( {\text{x}} \right)]{\text{e}}^{{{\text{j}}\upphi_{\text{y}} ({\text{x}})}}$$

The new image is then Fourier-transformed back to the spatial frequency domain where it is used to replace unknown data. The process is repeated and on the last iteration, (the number of iteration; 2) instead of substituting the new data into the data set from the previous iteration, a merging filter is used (McGibney et al. 1993).

**Fig. 1:**
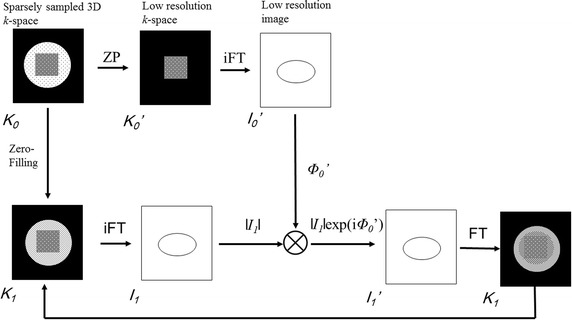
Flowchart of POCS reconstruction algorithm. Starting with the original sparsely sampled k-space data (*K*_0_), the central zone extended by zero filling (*K*_0_′) and reconstructed to make a low-resolution image (*I*_0_′) from which the low-frequency phase map is extracted (*φ*_0_′). The *K*0 data is also used to produce an initial estimate of the full *k*-space (*K*_1_), which is used to generate an estimate of the reconstructed image (I_1_). The magnitude of the reconstructed image estimate is then multiplied by exp(i*φ*_0_′) to produce a phase-corrected estimate (*I*_1_′) from which a new estimated k-space (*K*_1_′) is obtained. This k-space estimate is used to form a new k-space estimate (*K*_2_) by substituting missing values for all of the missing data in the original sparsely sampled data. The process repeats until the convergence criteria are met

Flowchart of POCS is shown in Fig. 1.

### Phantom study

The phantom studies were performed on a 1.5T scanner (Magnetom Avanto, Siemens, Erlangen, Germany). We scanned a resolution phantom (Multipurpose phantom, Siemens AG, Berlin, Germany). We acquired MRI scans with a 3D volumetric interpolated breath-hold examination (VIBE) gradient-echo sequence with fat-suppression. Chemical shift selective saturation (CHESS) was used to suppress fat signal intensity. Images were acquired in the transverse plane; the section thickness/interslice gap was 5/0 mm. The repetition time (TR, ms)/echo time (TE, ms) was 3.72/1.67, the flip angle (FA) 15°, the number of excitations 1, the field of view (FOV) 350 × 263 mm, and the matrix was 256 × 144. These sequence and parameters were the same as in our clinical hepatic MRI studies (protocol P1).

The same resolution phantom was scanned with a 3D VIBE gradient-echo sequence with fat-suppression using a PF image reconstruction (protocol P2), or PF with POCS algorithm (protocol P3). Chemical shift selective saturation (CHESS) was also used to suppress fat signal intensity. In protocol P2 and P3, we acquired data with 6/8 reduced Fourier space in the phase direction (off in the slice direction). In protocol P2, zero-filling was used for filling the missing truncated data of the k space. In protocol P2 and P3, reduced data of the k space were acquired in asymmetrical order.

### Clinical studies

This prospective study received institutional review board approval (registration number 799, “The efficacy of Gd-EOB-DTPA in the diagnosis of hepatobiliary diseases.”); prior informed consent was obtained from all patients. We did not receive any funding for this study.

Between June and December 2011, 55 consecutive patients (36 men, 19 women; mean age, 63.4 ± 12.2 years; age range, 25–82 years; mean weight, 57 kg; weight range, 39–78 kg), with known or suspected focal hepatic lesions were referred to our hospital for MRI study. These included HCC (n = 12), metastases of primary tumors (n = 3), and hemangioma (n = 6). And the other 34 patients had no focal hepatic lesions.

We acquired 3D hepatobiliary-phase MRI scans with a 3D volumetric interpolated breath-hold examination (VIBE) gradient-echo sequence with fat-suppression on a 1.5T scanner (Magnetom Avanto, Siemens, Erlangen, Germany). Chemical shift selective saturation (CHESS) was used to suppress fat signal intensity. The patients were injected intravenously with 0.025 mmol/kg (0.1 ml/kg) of EOB at a rate of 2 ml/s, followed by 40 ml saline at 2 ml/s using a power injector (Spectris Solaris EP, MEDRAD, Warrendale, PA). Images were acquired 15 min after the administration of EOB in the transverse plane; the section thickness/interslice gap was 5/0 mm. The repetition time (TR, ms)/echo time (TE, ms) was 3.72/1.67, the flip angle (FA) 15°, the number of excitations 1, the field of view (FOV) 350 × 263 mm, and the matrix was 256 × 144 (conventional method, protocol C1).

A generalized autocalibrating partially parallel acquisitions (GRAPPA) reconstructions method was included in this study (acceleration factor = 2). The data of the k-space were acquired in sequential order in the k_y_ direction and in centric order in the k_z_ direction. During the scans, the patients held their breath in end-expiration.

We also obtained hepatobiliary-phase imaes with either PF (protocol C2) or PF with POCS algorithm (protocol C3). In protocol C2 and C3, we acquired data with 6/8 reduced Fourier space in the phase direction (off in the slice direction). We acquired hepatobiliary phase images sequentially in the order of C1, C2, C3 images. The interval of these sequences were 10 s. In protocol C2, zero-filling was used for filling the missing truncated data of the k space. In protocol C2 and C3, reduced data of the k space were acquired in asymmetrical order. The acquisition time was 17-, 14-, and 14 s for protocols C1, C2 and C3, respectively.

### Qualitative analysis

*Phantom study* To assess image blurring of the peripheral phantom column among each protocols, we obtained subtraction images by subtracting P1- from P2 and P3 images (P2–P1, P3–P1) and compared these images.

*Clinical study* The MRI datasets of the 55 patients acquired under the 3 protocols were randomized and two readers, radiologists with 7 and 6 years of experience reading abdominal MRI and blinded to all patient data, performed qualitative analysis of peripheral vessel sharpness. We obtained 3 images from each patient using protocol C1, C2, and C3; this yielded 165 datasets. The radiologists graded the sets for vessel sharpness where grade 1 = most vessels blurred and indistinguishable from the hepatic parenchyma, grade 2 = most vessels blurred but distinguishable from the hepatic parenchyma, grade 3 = most vessels sharp with some blurring, and grade 4 = all vessels sharp at all points. We assessed all images of each data set for grading and compared the visual grading for vessel sharpness among the 3 protocols. In cases of interobserver disagreement, final decisions were reached by consensus. The degree of interobserver agreement for visual grading was determined by calculating the kappa value.

### Quantitative analysis

**Fig. 2:**
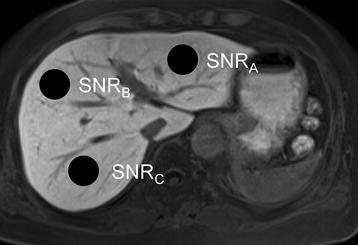
Quantification of SNR of the liver. We placed regions of interest (n = 3) on the left lobe, and on the anterior- and posterior compartment

The signal intensity (SI) of the hepatic parenchyma was measured by a radiologist with 7 years of experience reading abdominal MRI on the MRI console using observer-defined circular regions of interest (ROI) at the level of the hepatic hilum. The ROI were set on the left lobe and the anterior- and posterior compartments (Fig. 2). The signal-to-noise ratio (SNR) of the hepatic parenchyma was recorded as the average of the 3 ROI using the formula:

$${\text{SNR}} = {\text{SI}}\,\,{\text{of}}\,\,{\text{the-}}/{\text{SD}}\,\,{\text{of}}\,\,{\text{the}}\,\,{\text{hepatic}}\,\,{\text{parenchyma}}$$

The SNR in the SENSE-implemented sequence depends on acceleration and geometric factors. The latter vary over the image and describe local noise enhancements on the final reconstructed image; the background noise at parallel imaging thus varies with the spatial position (Pruessmann et al. 1999; Kurihara et al. 2002). Therefore we did not perform SNR calculations in a traditional way because of their unambiguous definition is difficult. We compared the SNR values among the 3 protocols.

### Statistical analysis

We compared the SNR of each techniques with the repeated one-way ANOVA. If there was a statistically significant difference among the different techniques, we performed pairwise comparisons using the paired *t* test with the Holm’s correction. For qualitative analysis, we used the Friedman’s test and the Wilcoxon signed-rank test with the Holm’s correction. Probability values of less than 0.05 were considered statistically significant. The scale for the kappa coefficients for interobserver agreement was: less than 0.20 = poor, 0.21–0.40 = fair, 0.41–0.60 = moderate, 0.61–0.80 = substantial, and 0.81–1.00 = near-perfect. Statistical analyses were with the free statistical software “R” (version 2.6.1; The R Project for Statistical Computing; http://www.r-project.org/).

## Results

### Qualitative analysis

**Fig. 3:**
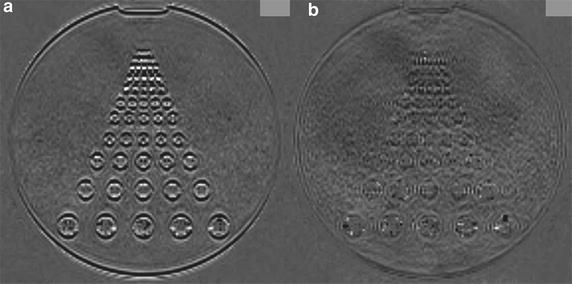
Subtraction images P2–P1 (**a**) and P3–P1 (**b**). Note the signal difference on the P2–P1 image on the peripheral phantom column. There is hardly any difference in SI on the P3–P1 image on the peripheral phantom column

*Phantom study* P2–P1 and P3–P1 images are shown in Fig. 3. The signal observed on the P2–P1 image in the peripheral phantom column is indicative of a difference in SI on P1 and P2 images and of blurring on the P2 image. The absence of a difference in SI on the P3–P1 image is evidence of minimal blurring.

**Fig. 4:**
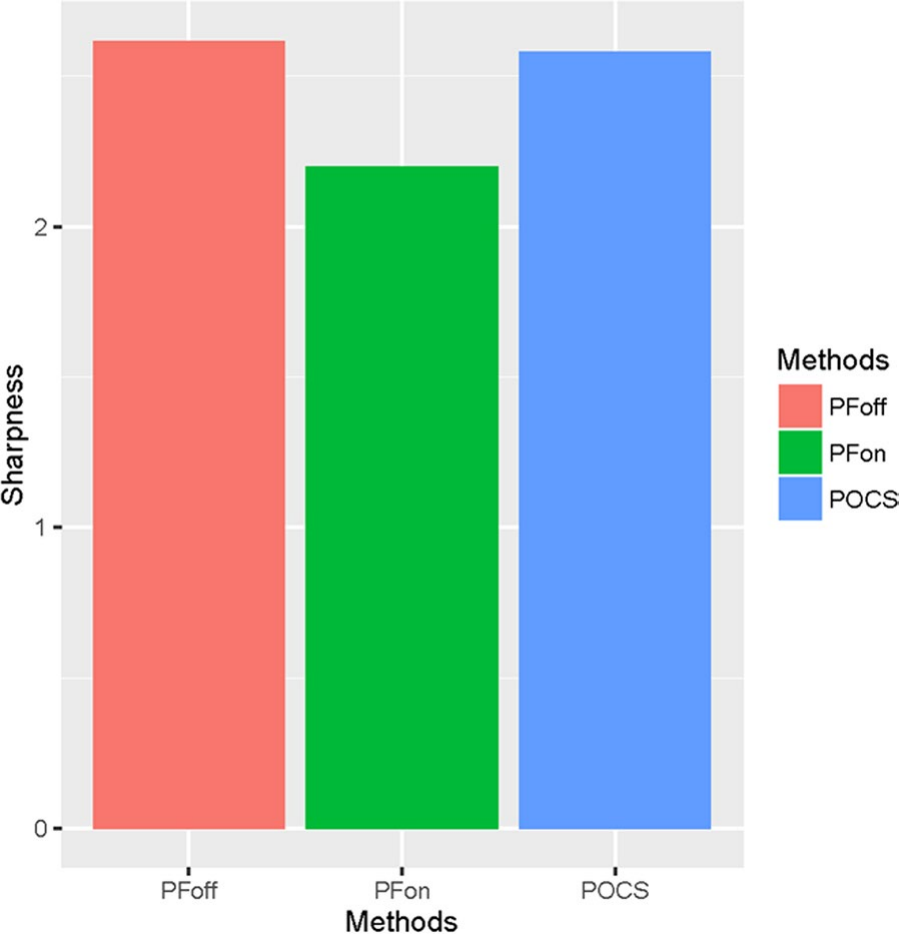
Visual grades assigned for vessel sharpness. The visual grading for vessel sharpness was 2.62 ± 0.49, 2.20 ± 0.40, 2.58 ± 0.49, respectively

*Clinical study* There is no acquisition failure from poor breath holding or other factors in 165 acquisitions. The visual grading for vessel sharpness was 2.62 ± 0.49, 2.20 ± 0.40, 2.58 ± 0.49, respectively (Fig. 4). The Friedman’s test showed a significant difference with respect to the visual grading for vessel sharpness among three groups (p < 0.01). Wilcoxon signed-rank test with the Holm’s correction showed a significant difference between C1- and C2-images (p < 0.01), and C2- and C3-images (p < 0.01). There was no significant difference between C1- and C3-images (p = 0.57). There was substantial interobserver agreement with respect to visual grading (kappa = 0.63).

**Fig. 5:**
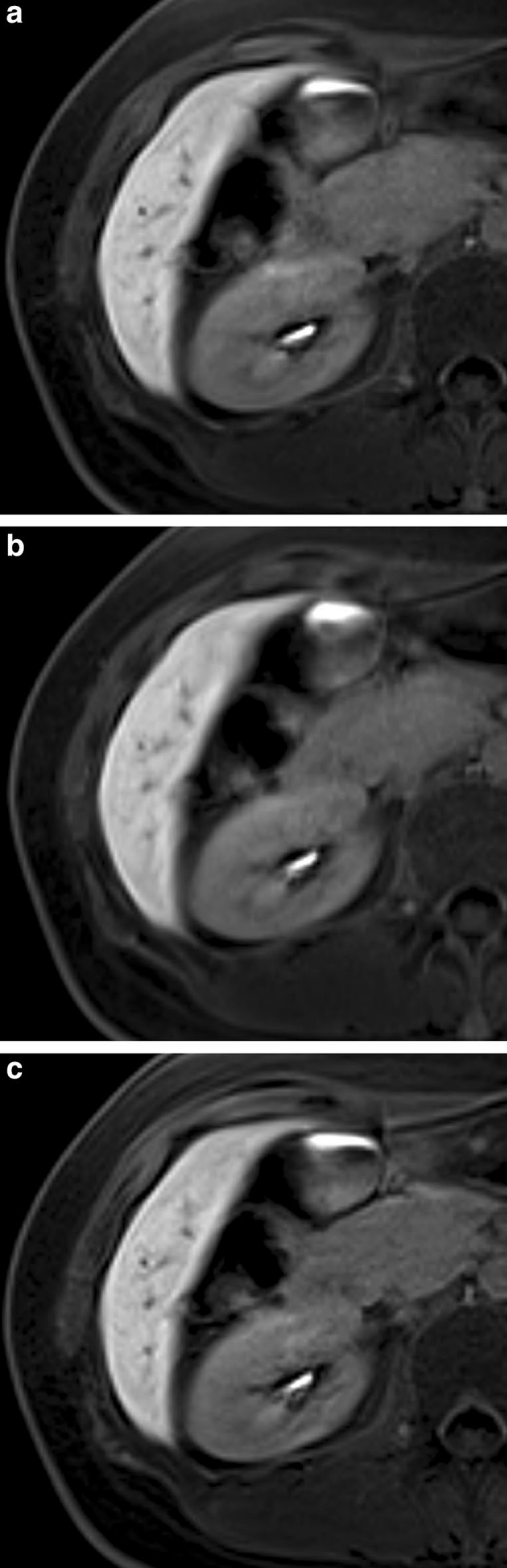
A 30-year-old woman who was suspected focal hepatic lesion. Her past history included breast cancer. **a** The hepatobiliary-phase imaging with conventional method. **b** The hepatobiliary-phase imaging with a PF algorithm. **c** The hepatobiliary-phase imaging with a PF with POCS algorithm. With conventional method (**a**) and with a PF with POCS algorithm (**c**), most vessels were sharp with some blurring (grade 3). But with a PF algorithm, most vessels blurred but distinguishable from the hepatic parenchyma (grade 2)

Images of representative cases are shown in Fig. 5.

### Quantitative analysis

**Fig. 6:**
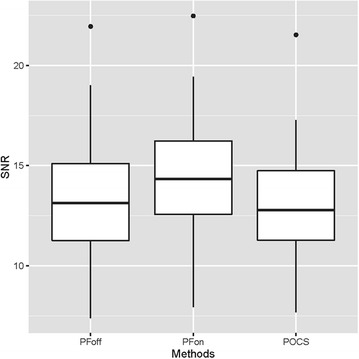
SNR of the hepatic parenchyma. SNR on C1-, C2-, and C3 images was 13.3 ± 2.67, 14.5 ± 2.80, and 13.1 ± 2.51, respectively

As shown in Fig. 6, the SNR of images C1, C2, and C3 was 13.3 ± 2.67, 14.5 ± 2.80, 13.1 ± 2.51, respectively. ANOVA showed a significant difference with respect to SNR among three groups (p = 0.02). Pairwise comparisons using the paired *t* test with the Holm’s correction showed a significant difference between C1- and C2-images (p < 0.01), and C2- and C3-images (p < 0.01). And there was no significant difference between C1- and C3-images (p = 0.19).

## Discussion

Our phantom- and clinical studies suggest that the POCS algorithm is useful for reducing the acquisition time without deterioration of the image quality on 3D volumetric interpolated gradient-echo sequences used in hepatic MRI. This technique significantly improved the vessel sharpness of PF images. In addition, there were no significant differences in image sharpness and SNR on images acquired with PF with POCS and images obtained without PF. Our findings suggest that PF with POCS imaging may be an alternative to conventional imaging at 17 % shorter scan time.

PF has been used to reduce the scan time mainly at dynamic contrast-enhanced MRI (Perman et al. 1996). Its disadvantage, image blurring, can be improved. Madore (2002) showed that unaliasing by Fourier-encoding the overlaps in the temporal dimension (UNFOLD) reduces artifacts on PF images. Chen et al. (2008) reported that multi-scheme PF reconstruction method could reduce artifacts. Lee and Munson (2000) who used spatially variant apodization (SVA) for image reconstruction from PF data found that this reduced blurring. Our results supported the finding that PF reduces the scan time and suggest that it is useful for mitigating image blurring.

Singh et al. (2004) assessed the feasibility of using two-dimensional PF with POCS to reduce the acquisition time at MRI of coronary arteries. They concluded that PF with POCS is a potentially useful technique for reducing the acquisition time and improving spatial resolution at breath-hold coronary MRA. Raj et al. (2006) who investigated whether the POCS algorithm filters out motion artifacts on time-series two-dimensional MR digital subtraction angiographs concluded that a POCS algorithm can be constructed that imposes consistency constraints on dynamic MRA data to filter out motion artifacts while preserving vascular enhancement.

We found that the image quality yielded by PF with POCS was similar to that obtained without PF and that the scan time was reduced by 17 % without a reduction in SNR, and without an increase in artifacts. So we believe that shortening scan time yielded lightening of patient’s burden. In our phantom study there was no difference in SI on the peripheral phantom column on the subtraction images obtained by subtracting the image without PF from the image with PF with POCS. This suggests that there was minimal blurring on the PF with POCS image. In our clinical study, SNR was significantly higher on images acquired with the PF- than the conventional method, possibly because PF increases the effective slice thickness. Although POCS reduced SNR, there was no significant difference between the SNR on images obtained using PF with the POCS algorithm and SNR on images acquired with the conventional method, because the increase in SNR by PF compensated for the SNR decrease by POCS. Our results showed that PF with POCS is useful not only with respect to the visualization of vascular structures but also at Gd-EOB-DTPA-enhanced hepatobiliary-phase hepatic MRI. Although we believe this method is useful for arterial-phase and portal-phase hepatic MRI, further studies need to be conducted to evaluate the usefulness of PF with POCS algorithm in arterial-phase and portal-phase hepatic MRI.

Our study has some limitations. First, we did not compare our 3 protocols for the detection of HCC. Second, we scanned the patients under breath holding at end-expiration and did not investigate the usefulness under free-breathing conditions. Third, we reduced the scan time by 17 % when we applied the POCS algorithm, a change may be relatively small. Fourth, as we applied 3 scanning protocols consecutively, the patients’ expiration may have been different. Fifth, three images were reconstructed from different acquired data (three times of acquisition in hepatobiliary phase) by three methods not treating one same data by three different methods. So the comparison among the three images was based not only the difference of three reconstruction methods but also the possible difference of acquired data, which possibly contaminated by minimal motion artifact or other factors.

## Conclusion

We suggest that the POCS algorithm is useful at Gd-EOB-DTPA-enhanced hepatobiliary-phase MRI because it facilitates shortening the scan time by 17 % without a decrease in SNR an increase in artifacts.
